# Prosystemin overexpression induces transcriptional modifications of defense-related and receptor-like kinase genes and reduces the susceptibility to *Cucumber mosaic virus* and its satellite RNAs in transgenic tomato plants

**DOI:** 10.1371/journal.pone.0171902

**Published:** 2017-02-09

**Authors:** Giovanni Bubici, Anna Vittoria Carluccio, Livia Stavolone, Fabrizio Cillo

**Affiliations:** 1 Istituto per la Protezione Sostenibile delle Piante, Consiglio Nazionale delle Ricerche, Bari, Italy; 2 International Institute of Tropical Agriculture, Ibadan, Oyo State, Nigeria; Texas A&M University College Station, UNITED STATES

## Abstract

Systemin is a plant signal peptide hormone involved in the responses to wounding and insect damage in the *Solanaceae* family. It works in the same signaling pathway of jasmonic acid (JA) and enhances the expression of proteinase inhibitors. With the aim of studying a role for systemin in plant antiviral responses, a tomato (*Solanum lycopersicum*) transgenic line overexpressing the *prosystemin* cDNA, i.e. the systemin precursor, was inoculated with *Cucumber mosaic virus* (CMV) strain Fny supporting either a necrogenic or a non-necrogenic satellite RNA (satRNA) variant. Transgenic plants showed reduced susceptibility to both CMV/satRNA combinations. While symptoms of the non-necrogenic inoculum were completely suppressed, a delayed onset of lethal disease occurred in about half of plants challenged with the necrogenic inoculum. RT-qPCR analysis showed a correlation between the systemin-mediated reduced susceptibility and the JA biosynthetic and signaling pathways (e.g. transcriptional alteration of *lipoxygenase D* and *proteinase inhibitor II*). Moreover, transgenically overexpressed systemin modulated the expression of a selected set of receptor-like protein kinase (RLK) genes, including some playing a known role in plant innate immunity. A significant correlation was found between the expression profiles of some RLKs and the systemin-mediated reduced susceptibility to CMV/satRNA. These results show that systemin can increase plant defenses against CMV/satRNA through transcriptional reprogramming of diverse signaling pathways.

## Introduction

Systemin is a plant signal peptide hormone involved in the wound response and insect damage in the *Solanaceae* family [1, 2]. This 18 amino acids peptide is released from the C-terminal region of a larger precursor of 200 amino acids, called prosystemin [3]. Once perceived by the systemin cell-surface receptor kinase SR160 [4, 5], systemin activates the expression of protease inhibitors (PIs) [2, 3, 6, 7]. Several genetic studies using tomato (*Solanum lycopersicum*) mutants suggested that systemin works in the same signaling pathway of jasmonic acid (JA) (reviewed by [1]). A model for the systemin signaling pathway has been previously proposed by Schilmiller and Howe [8].

As a consequence of the cross-talk among phytohormones, the systemin-signaling has also implications for an array of defense genes not directly related to JA [9]. Systemin-signaling cascade antagonizes pests and renders plants more attractive for parasitoids [10]. In transgenic plants overexpressing the prosystemin cDNA, an increased resistance against insect pests such as *Macrosiphum euphorbiae*, *Spodoptera littoralis* and *Manduca sexta*, and necrotrophic fungal pathogens including *Botrytis cinerea* and *Alternaria solani* has been observed [9, 11–14]. In addition, systemin can enhance plant tolerance against necrotrophic fungal pathogens and salt stress [9, 11, 12, 15].

Although it is known that plants possess phytohormone-mediated resistance mechanisms against pathogens, including viruses, no research has been conducted so far to explore the role of systemin against viral diseases [16, 17]. A role of systemin in plant antiviral responses was supported by a previous transcriptomic analysis showing a prosystemin overexpression in tomato plants infected by *Cucumber mosaic virus* (CMV) associated with a necrogenic variant of its satellite RNA (satRNA; Cillo F., unpublished).

CMV (genus *Cucumovirus*, family *Bromoviridae*) is a tripartite plant virus with four or five encapsidated positive single-strand RNAs. Transmitted in a non-persistent manner by over 80 species of aphids, it is present worldwide and can infect over 1200 plant species belonging to about 100 botanical families of monocots and dicots, including vegetables, ornamentals, woody and semi-woody plants [18]. Some strains of CMV can support a satRNA, which can affect considerably the virus (helper) replication, pathogenesis and symptom development on the plant. In fact, the so-called benign variants of satRNA attenuate drastically the symptoms typically induced by CMV helper virus whereas the necrogenic variants largely exacerbate the symptoms, inducing extensive cell death [19–22]

With the aim of elucidating the molecular mechanisms that may network the systemin signaling with antiviral defenses and necrosis development, we evaluated the *prosystemin* overexpression effects on the susceptibility of tomato to CMV/satRNA, and analyzed the transcriptomic changes on a set of stress hormone and receptor-like kinase (RLK) genes. Our data shed new light on the systemin-mediated defense network, and show functional implications for diverse molecular pathways of tomato-virus interaction.

## Materials and methods

### Plant material, virus and inoculation

Seedlings of tomato were grown in pots in a growth chamber at 24±2°C under 16 h light/8 h dark cycle. The cv. Moneymaker, the cv. Better Boy (BB) and the corresponding transgenic line overexpressing the prosystemin gene (BBP+), were used in the described experiments [6]. The aggressive isolate CMV-Fny, belonging to the subgroup IA [23], was used in combination with the necrogenic satRNA variant 77-satRNA (GenBank accession no. X86422) to form the necrosis-inducing CMV/satRNA combination (S1 Fig) here termed ‘FN’ [22]. Additionally, we developed a non-necrogenic mutant of 77-satRNA designated ‘NNmut-satRNA’ which, in association with CMV-Fny, formed the inoculum here termed ‘FNNmut’. The infection by FNNmut induced growth stunting, mosaic and leaf malformation but not necrosis (S1 Fig). In order to obtain NNmut-satRNA, three nucleotide positions were substituted (G284A, T289G and C291T) within the so-called necrogenic domain [24], using the QuikChange XL site-directed mutagenesis kit (Stratagene, La Jolla, CA), following the manufacturer’s instructions (S2 Fig).

CMV/satRNA combinations were inoculated according to published procedures [22]. Fourteen plants (biological replicates) per each plant genotype/inoculum combination were used.

Viral symptoms were visually evaluated at 9, 16, 21 and 28 days post-inoculation (dpi), according to a 0 to 3 arbitrary scale, where 0 = no symptoms; 1 = growth reduction and leaf distortion; 2 = systemic development of necrotic lesions on stems and leaves; and 3 = dead plant (see also S1 Fig. for definition of disease symptoms). Virus titer, expressed as accumulation of RNA2 of CMV in the tissues, was measured at 9 dpi with real-time quantitative reverse transcription PCR (RT-qPCR; see below) using the primer pair CMV_RNA2 (S1 Table).

### RNA extraction and real-time reverse transcription—quantitative PCR

Total RNA was extracted using TRIzol^®^ Reagent, according to the manufacturer’s instructions (Life Technologies, Carlsbad, CA), from the second true leaf (100 mg of leaf tissue) sampled at 9 dpi from six representative plants per each plant genotype/inoculum combination. Reverse transcription and real-time quantitative PCR reactions were performed as previously described [25]. Briefly, reactions containing 10 ng of cDNA 400 nM of each forward and reverse primers and Fast SYBR^®^ Green Master Mix (Applied Biosystems, Foster City, CA) were assembled in a total volume of 12.5 μL. Two technical replicates per sample were used. The reactions were conducted in a CFX96 Touch^™^ Real-Time PCR Detection System (Bio-Rad Laboratories, Hercules, CA) using the following cycling profile: 10 s at 95°C, followed by 40 cycles of 3 s at 95°C and 30 s at 60°C, as recommended by Applied Biosystems. Data obtained were converted into relative gene expression using the 2^-ΔΔCT^ method [26] corrected for the PCR efficiency of each amplicon using Bio-Rad CFX Manager 3.1 software (Bio-Rad Laboratories).

Primers for plant and viral genes employed in RT-qPCR assays are listed in S1 Table. They were retrieved from the literature or designed for this study using PrimerQuest software (Integrated DNA Technologies, Inc.; http://eu.idtdna.com/Primerquest/Home/Index). The ubiquitin 3 (*ubi3*) mRNA was used as the reference gene, since its expression levels have been found unaltered in tomato plants infected by different viruses [25].

### Data analysis

Relative normalized expression values deriving from RT-qPCR experiments were subjected to the analysis of variance (ANOVA) after a log-transformation aimed to fulfill data distribution normality and homoscedasticity. Multiple pairwise comparisons were made by the Fisher’s least-significant-difference test (LSD; *P*<0.05). Statistical analysis was done using SAS 9.0 (SAS Institute, Cary, NC).

## Results

### *Prosystemin* gene expression is up-regulated by CMV/satRNA infections

**Fig 1:**
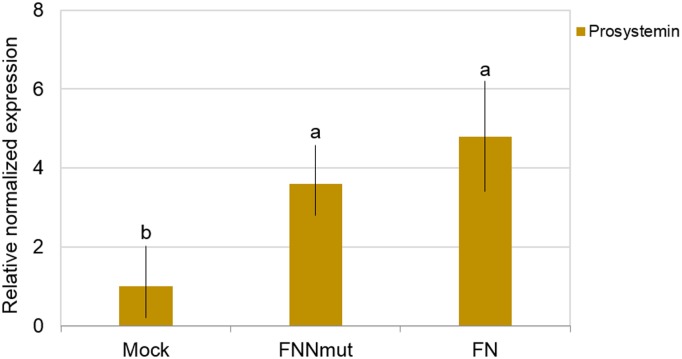
*Cucumber mosaic virus*/satellite RNA infections alter *prosystemin* expression in tomato plants. Expression of *prosystemin* transcript measured with RT-qPCR in tomato plants cv. Moneymaker at 9 days post-inoculation with *Cucumber mosaic virus* (CMV-Fny) in combination with its satellite RNAs, either necrogenic (FN) or non-necrogenic mutant (FNNmut). Gene expression is relative to mock and normalized by *ubiquitin* gene (*ubi3*). Bars on the columns represent the standard deviation (n = 6). Columns with different letters are significantly different according to LSD test (P<0.05).

In a previous transcriptomic analysis, *prosystemin* transcript resulted approximately three-fold overexpressed in tomato plants infected by *Cucumber mosaic virus* (CMV) in combination with 77-satRNA (i.e., FN), compared to mock-inoculated plants. In order to validate that observation, in this study we inoculated tomato cv. Moneymaker plants with FN or FNNmut. In systemically infected leaves sampled from the second node above the cotyledons, immediately before the necrosis onset at 9 dpi, RT-qPCR analysis revealed an expression of *prosystemin* in FNNmut- or FN-inoculated plants increased by 3.6 or 4.8 fold, respectively, compared to mock-control (Fig 1). This result confirms the responsiveness of *prosystemin* in compatible interactions between tomato and CMV/satRNAs.

### Transgenic *prosystemin* overexpression reduces the susceptibility of tomato to CMV/satRNA

To further investigate the possible functional role of *prosystemin* in the tomato-CMV/satRNA pathosystem, a 35S::*prosystemin* transgenic tomato line (BBP+) and its wild type counterpart, cv. Better Boy (BB), were inoculated with FN or FNNmut.

**Fig 2:**
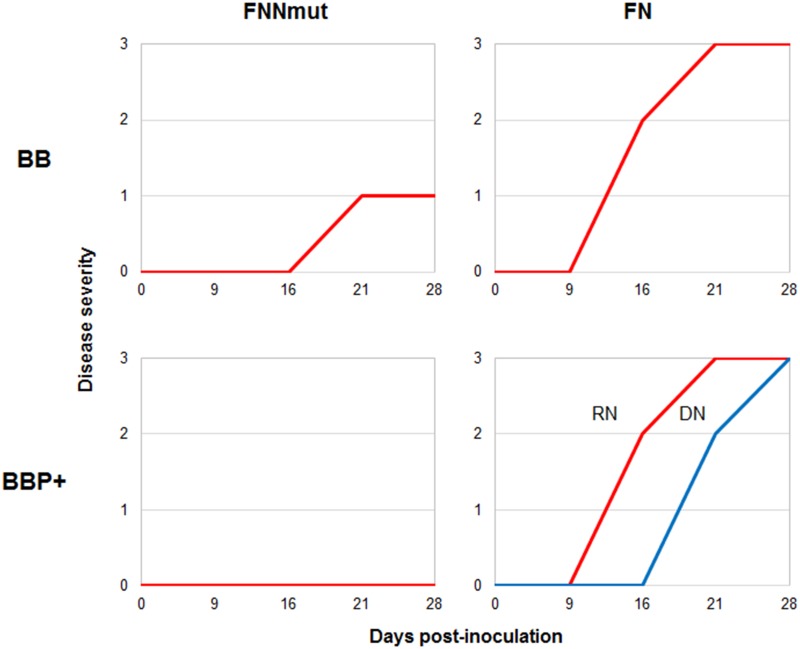
Severity of disease symptoms induced by *Cucumber mosaic virus*/satellite RNA infections is reduced in transgenic tomato plants overexpressing *prosystemin*. Severity of symptoms caused by *Cucumber mosaic virus* (CMV) combined with two satellite RNAs, either necrogenic (77-satRNA) or non-necrogenic (the mutant variant NNmut-satRNA), on tomato plants cv. Better Boy, wild type (BB) or overexpressing the *prosystemin* transgene (BBP+). In each graph, the disease severity scores observed on 14 plants is shown. In the BBP+/FN graph (bottom right), two groups of plants that displayed significantly different disease development are indicated with a red (8 plants showing rapid necrosis or RN) and a blue (6 plants showing delayed necrosis or DN) line.

In cv. BB, typical symptoms induced by FN or FNNmut developed over the period of observation (28 days). In all BB plants inoculated with FN, systemic necrosis appeared on stems, petioles and leaves at 16 dpi, and plant death occurred at 21 dpi. The BB plants inoculated with FNNmut showed a moderate growth reduction, mild mosaic and leaf distortion at 21 dpi, and the same symptoms were still evident at 28 dpi (Fig 2, S1 Fig).

**Fig 3:**
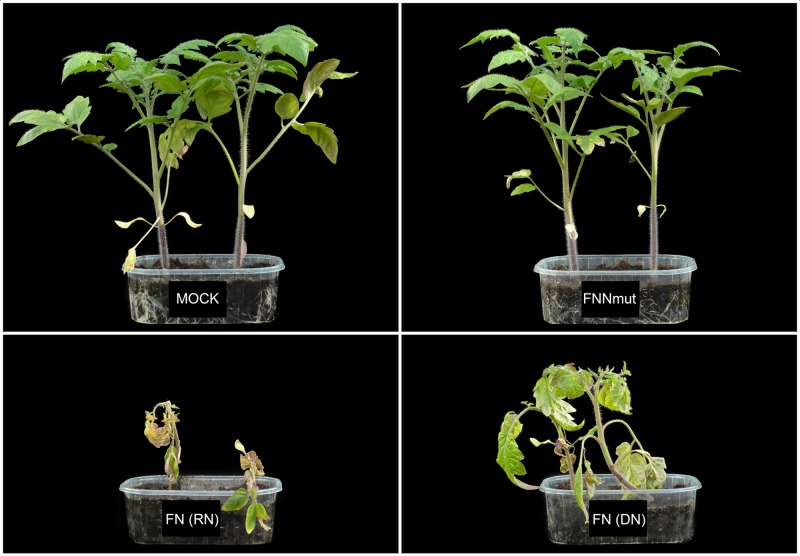
Disease symptoms induced by *Cucumber mosaic virus*/satellite RNA infections in transgenic tomato plants overexpressing *prosystemin*. Disease phenotypes in transgenic plants cv. Better Boy overexpressing *prosystemin* (BBP+) 21 days post-inoculation with CMV/satRNA combinations. Top Left panel, mock-inoculated healthy control (Mock); Top Right, plants inoculated with CMV/NNmut-satRNA showing suppression of symptoms (FNNmut); Bottom Left, plants inoculated with CMV/77-satRNA showing rapid and lethal necrosis [FN (RN)]; Bottom Right, plants inoculated with CMV/77-satRNA showing partial resistance and delayed necrosis [FN (DN)];

On the other hand, in BBP+ plants, such symptom development was significantly altered (Figs 2 and 3). Remarkably, a complete suppression of typical symptoms occurred in BBP+ plants inoculated with FNNmut (Figs 2 and 3). A group of 8 out of 14 plants inoculated with FN showed the typical progress of lethal disease, with onset of symptoms at 16 dpi and rapid necrosis (RN) and plant death at 21 dpi, whereas the remaining six plants showed at the same time point an attenuated and delayed necrosis (abbreviated hereafter as DN) (Figs 2 and 3). The latter group of plants, however, died by 28 dpi showing a 7-day delay in the lethal outcome. These two groups of FN-infected transgenic plants, showing at 21 dpi either the RN or the DN phenotype, were analyzed separately across the entire study. The experiment was repeated three times, and every time approximately a half of the BBP+ plants inoculated with FN exhibited a DN phenotype, while FNNmut-inoculated BBP+ (BBP+/FNNmut) plants remained symptomless.

**Fig 4:**
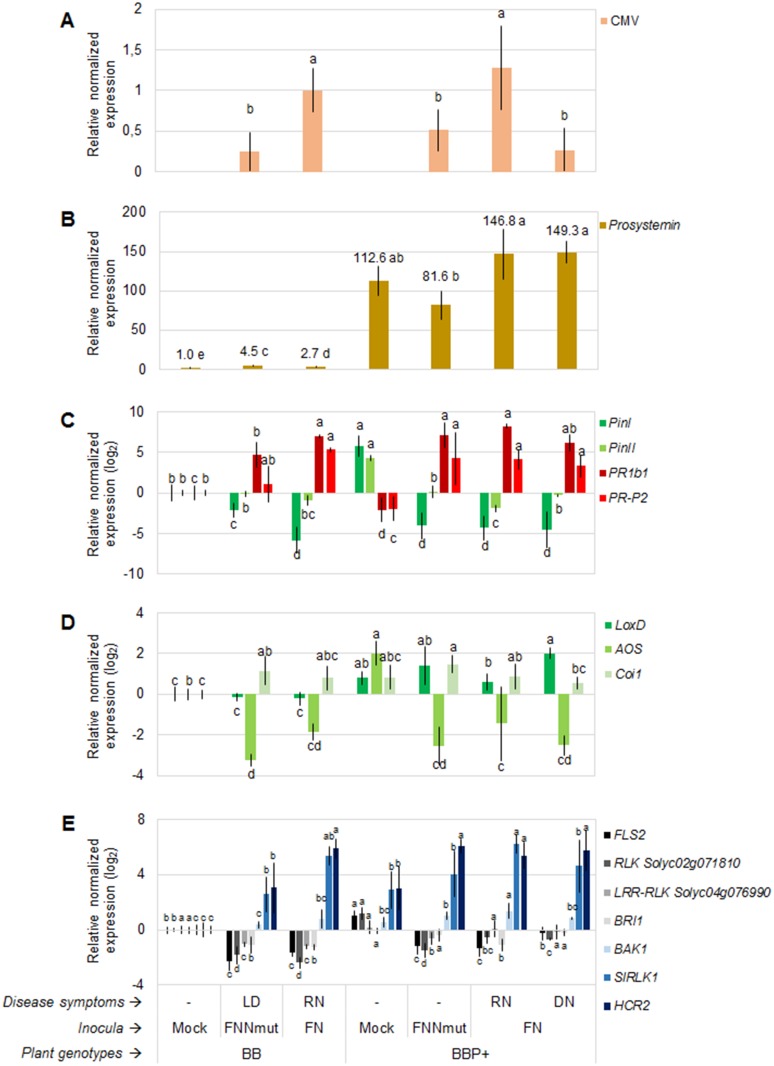
Both ectopic overexpression of *prosystemin* and *Cucumber mosaic virus*/satellite RNA infections trigger extensive transcriptional reprogramming. Gene expression measured with RT-qPCR in tomato plants cv. Better Boy, wild type (BB) or overexpressing the *prosystemin* transgene (BBP+), at 9 days post-inoculation with *Cucumber mosaic virus* (CMV) in combination with the necrogenic 77-saRNA (FN) or the non-necrogenic mutant NNmut-satRNA (FNNmut). A: *prosystemin*; B: CMV titer, expressed as RNA2 accumulation in leaf tissues, and relative to BB/FN plants; C: marker-genes of salicylate and jasmonate signaling pathways; D: jasmonate-biosynthesis genes; E: receptor-like protein kinases. Gene expression is relative to mock-inoculated BB plants, and normalized by *ubiquitin* gene (*ubi3*). Bars on the columns represent the standard deviation (n = 6). Per each individual gene analyzed separately, columns with different letters are significantly different according to LSD test (*P*<0.05). Disease symptoms: ‘-‘ = no symptoms; LD = leaf distortion; RN = rapid necrosis; DN = delayed necrosis.

The viral titer at 9 dpi, expressed as RNA2 accumulation in systemically infected leaves, was significantly lower in FNNmut-inoculated plants than in FN-inoculated ones, both in the BB and BBP+ genotype, indicating a reduced rate of replication and/or translocation in plant tissues of CMV supporting the mutant satRNA (Fig 4A). The titer of FNNmut-RNA2 was statistically comparable in BB versus BBP+ plants, where accumulation levels showed either 0.2 or 0.5 fold change (fc), respectively, compared to RNA2 levels in FN-inoculated BB plants (BB/FN), despite the mutant satRNA-induced symptoms were suppressed in the transgenic line. The titer of CMV RNA2 was not significantly different between the wild type and transgenic tomato lines showing RN (1 vs. 1.3 fc, respectively), but was significantly lower in BBP+/FN plants showing DN (0.3 fc).

The *prosystemin* transcript resulted 4.5 or 2.7-fold overexpressed in BB plants inoculated with FNNmut or FN, respectively, compared to mock-inoculated BB (Fig 4B). In BBP+ plants, where expression of the transgene accounted for an approximately 100-fold increased accumulation of *prosystemin*, a further induction of this gene occurred in plants inoculated with FN, but not in those inoculated with FNNmut.

Collectively, these results indicate that transgenic overexpression of systemin in tomato reduces the susceptibility to CMV/satRNA infections, as it suppresses the symptoms induced by FNNmut and partially attenuates the effects of necrosis induced by FN. In BBP+, the DN phenotype associated to some FN-infected plants, but not the symptomless phenotype in FNNmut-infected plants, correlated with a significant decrease in viral RNA levels.

### Systemin-mediated induction of jasmonate-signaling pathway is counteracted by virus-mediated enhancement of salicylate-signaling pathway in infected plants

Based on the observation of *prosystemin* overexpression in CMV/satRNA-infected plants and the knowledge that systemin triggers plant defense responses, we investigated the perturbation of specific signal transduction mechanisms upon CMV/satRNA infections in BB and BBP+ plants by RT-qPCR analysis of four genes typically induced by either JA signaling (proteinase inhibitors *PinI* and *PinII*), or salicylic acid (SA) signaling (pathogenesis-related proteins *PR1b1* and *PR-P2*) (Fig 4C). As expected, *PinI* and *PinII* were substantially up-regulated by 4–5 fc in transgenic, non-infected plants. On the other hand, the viral infection down-regulated *PinI* expression by -2.1 to -5.8 log_2_ fold change (l_2_fc) in BB and BBP+ plants, respectively, and *PinII* expression only in inoculated compared to healthy BBP+. *PinII* resulted significantly down-regulated in BBP+ plants showing RN (-1.9 l_2_fc) compared to those showing DN (-0.3 l_2_fc). In contrast, the *PR1b1* and *PR-P2* levels were suppressed (-2.1 and -1.9 l_2_fc, respectively) in healthy transgenic plants, but were substantially up-regulated by viral infection both in BB (4.7 to 7 l_2_fc and 1 to 5.3 l_2_fc, respectively) and in BBP+ plants (6.2 to 8.2 l_2_fc and 3.3 to 4.2 l_2_fc, respectively).

The *lipoxygenase D* (*LoxD*) gene, encoding one of the most upstream enzymes in the JA biosynthetic pathway, was up-regulated by 0.8 l_2_fc in BBP+/mock compared to BB/mock plants (Fig 4D). Interestingly, as for *PinII*, its expression in BBP+ plants showing RN (0.6 l_2_fc) *LoxD* was significantly lower than in those showing DN (2 l_2_fc).

*Allene oxide synthase* (*AOS*), which acts downstream of *LoxD*, showed an expression profile in good correlation with that of *PinI*, being up-regulated in non-infected transgenic plants (2 l_2_fc), but down-regulated by the viral infections both in BB (-1.9 to -3.3 l_2_fc) and BBP+ (-1.5 to -2.5 l_2_fc). On the contrary, *Coronatine-insensitive 1* (*Coi1*), which acts downstream of JA synthesis, was unaffected by *prosystemin* overexpression (0.84 l_2_fc), but up-regulated by viral infection (0.8–1.5 l_2_fc), except in BB/FN and BBP+/FN plants showing DN. Therefore, differential gene expression of *PinII* and *LoxD*, but not of *AOS* and *Coi1*, in BBP+/FN plants showing RN versus DN indicated a possible correlation between alterations in the JA pathway and the reduced susceptibility to necrogenic FN infections. The reproducibility of these gene expression profiles were tested and confirmed in three independent experiments.

### Transgenic *prosystemin* overexpression affects RLKs transcriptional response, and modulation of some RLKs correlates with the reduced susceptibility to CMV/satRNA in transgenic plants

Cell membrane RLKs activates signal cascades once external stimuli are perceived. To further study the role of systemin in CMV/satRNA-tomato interaction, we investigated the expression profiles of seven tomato RLKs, of which some known for participating in the PTI defense mechanisms, and others never characterized before.

Four out of the seven RLKs analyzed, namely *FLS2*, *RLK-Solyc02g071810*, *LRR-RLK-Solyc04g076990* and *BRI1*, were overall down-regulated by infection of both FNNmut and FN, in contrast with *SlRLK1* and *HCR2-0A*, which were up-regulated in infected plants, compared to the corresponding healthy controls. Only BAK1 showed no expression variations upon viral infections in BB plants.

As for the responsiveness of the selected RLKs to *prosystemin*, *FLS2* (1 l_2_fc), *RLK-Solyc02g071810* (1.2 l_2_fc), *SlRLK1* (2.9 l_2_fc) and *HCR2-0A* (3 l_2_fc), were up-regulated in the transgenic mock-inoculated plants, where the expression of *LRR-RLK-Solyc04g076990* (0.2 l_2_fc), *BRI1* (-0.1 l_2_fc) and *BAK1* (0.6 l_2_fc) was substantially unaltered, compared to mock-inoculated BB (Fig 3E).

CMV/satRNA infections reduced the expression of *FLS2* in both BB (-1.6 and -2.3 l_2_fc in FNNmut and FN, respectively) and BBP+ genotype (-0.2 to -1.4 l_2_fc), compared to the corresponding healthy controls (0 and 1 l_2_fc, respectively). BBP+ plants showing RN (-0.2 l_2_fc) and DN (-1.4 l_2_fc) showed a significantly different *FLS2* expression. Similarly, *RLK-Solyc02g071810*, here selected for gene expression profiling because resulted responsive to CMV infections in a previous study, was suppressed in all inoculated BB (-1.8 l_2_fc to -2.4 l_2_fc in FNNmut and FN, respectively) and BBP+ (-0.6 to -1.5 l_2_fc), compared to the corresponding controls (0 and 1.2 l_2_fc, respectively). A very moderate down-regulation of this gene occurred in BBP+/FN, with no difference between RN (0.6 l_2_fc) and DN phenotypes (0.7 l_2_fc). *LRR-RLK-Solyc04g076990*, another CMV-responsive RLK, was also down-regulated by both CMV/satRNA inoculations in non-transgenic plants (-1 to -1.2 l_2_fc in FNNmut and FN, respectively), but only by FNNmut (-0.6 l_2_fc) in the transgenic genotype. In BBP+/FNNmut plants, where the virus symptoms were completely suppressed, the down-regulation of this LRR-RLK was less pronounced than in BB plants challenged with the same inoculum (-0.6 vs. -1.2 l_2_fc).

A more moderate response to CMV/satRNA occurred for two functionally associated and PTI-involved genes, *BRI1* and *BAK1*. *BRI1* was down-regulated in BB infected plants (-1.1 and-1.3 l_2_fc in FNNmut and FN, respectively) as well as in BBP+/FN plants showing RN (-1 l_2_fc), but unaltered in the remaining genotype/inoculum combinations. There was a significant difference in *BRI1* levels between BB/FNNmut (-1.1 l_2_fc) and BBP+/FNNmut (-0.4 l_2_fc). In contrast, *BAK1* tended to be up-regulated by the viral infections, although a significant overexpression occurred only in BBP+/FN plants showing RN (1.4 l_2_fc), as compared to BBP+/FN showing DN (0.8 l_2_fc).

*SlRLK1*, the tomato orthologue of *CaRLK1*, in our experiments showed significantly higher expressions in inoculated (2.6 to 5.3 l_2_fc in FNNmut and FN, respectively) than non-inoculated BB plants, and in BBP+ plants showing RN (6.3 l_2_fc) than in non-inoculated BBP+ (2.9 l_2_fc) or those showing DN (4.7 l_2_fc). The virus infection also induced the homologue of the *Cladosporium* resistance gene *Cf-2* (*HCR2-0A*) in BB (3 to 5.9 l_2_fc in FNNmut and FN, respectively) and BBP+ (5.4 to 6 l_2_fc).

In summary, the suppression of FNNmut symptoms in BBP+ was associated with significant variations of *LRR-RLK-Solyc04g076990* and *BRI1* expression. The different RN and DN phenotypes occurring in BBP+/FN plants correlated with significantly different expression levels of *FLS2*, *BRI1*, *BAK1* and *SlRLK1*.

## Discussion

The infection by CMV-Fny in combination with the necrogenic 77-satRNA (FN) induces a rapid and lethal necrotic disease in tomato (RN phenotype). We had observed that such infection induced, among other responses, a *prosystemin* enhanced expression, and therefore investigated the possible role of systemin in the compatible interactions between tomato and CMV/satRNA.

Our results indicate that the transgenic overexpression of *prosystemin* significantly reduces the susceptibility to FN-induced lethal necrosis in about 50% of the plants, and completely suppresses symptoms caused by FNNmut. The reasons for which only a half of FN-infected BBP+ plants displayed DN are unclear, and may depend on the genetic background of the transgenic tomato line. In fact, BB is an hybrid genotype and BBP+ has been stabilized by back-crossing for transgene homozygosity, but the possibility that other single or multiple host genes concurring to the partial resistance are still in a heterozygous condition cannot be ruled out.

The attenuated necrosis, i.e. the observed 7 day-delayed onset of the lethal disease, was associated with a significantly lower titer of CMV. On the other hand, FNNmut symptoms suppression did not correlate with a reduction of viral accumulation level. This observation is in concordance with previous studies proving that the severity of symptom expression is not always correlated to viral accumulation in infected tissues (reviewed in [27, 28]). Therefore, different mechanisms either implying or not suppression of viral accumulation likely underlie the interactions of tomato with the two CMV/satRNA combinations. Taken together, this scenario reveals the reduced susceptibility of tomato to a viral disease due to the ectopic *prosystemin* overexpression and the enhanced systemin signaling.

Systemin works in the same signaling pathway of JA [1] and also affects other phytohormones [9]. Consistently, BBP+ plants showed an increased transcription of genes both in the biosynthesis (*LoxD*, *AOS*) and signal transduction pathways of JA (*PinI* and *PinII*), as well as a down-regulation of SA-dependent genes such as *PR1b1* and *PR-P2*. In fact, it is well known that JA and SA pathways are antagonistically inter-connected. Overall, and with exceptions, JA activates defenses against insect pests, wounding and necrotrophic fungal pathogens, whereas SA induces defenses against biotrophic pathogens and viruses [16, 28–32]. Also, a balance between endogenous SA and JA plays a key role for determining the degree of resistance [33]. At present, a role for JA signaling in plant defense against viruses has not been unequivocally established. Examples include the work of Ryu and colleagues [34], who demonstrated that a rhizobacterial strain provided systemic protection against CMV in Arabidopsis following a signaling pathway independent of SA but dependent on JA and correlating with overexpression of JA-dependent genes. Additionally, it was found that exogenous application of JA reduced Arabidopsis susceptibility to CMV infections, and that even higher inhibitory efficiency was obtained when JA was followed by a SA treatment [35]. Similarly, our data indicate that a systemin-driven signal activates the JA defense pathway and primes (*sensu* [36]) transgenic plants for increased resistance to the CMV infections. Upon viral infection, expression profiles of SA-dependent genes increases and that of JA-dependent genes decreases, generating a new balance between the two defense pathways as typically found in plant-virus compatible interactions [29, 37]. Nevertheless, reduced susceptibility against both FN and FNNmut correlates with the enhanced expression of *LoxD* in BBP+ plants. This finding is in agreement with the view that decreased activity of *Lox* genes is associated with enhanced susceptibility to viruses in plants [38, 39]. At least in one case, overexpression of *Lox* genes and a general up-regulation of oxylipin-biosynthesis genes were associated with more severe, necrogenic plant-virus compatible interactions [40]. The same research group, however, showed that *Coi1* silencing in *Nicotiana benthamiana* accelerated cell death induced by *Potato virus X* expressing a potyviral helper component-proteinase, leading to the conclusion that reduced JA perception correlated with increased necrosis symptoms [41]. The different hosts, viruses and tissues analyzed, the complexity and redundancy of the *Lox* gene family, the timing of gene expression analysis may all account for these divergent outcomes that, however, reinforce the general notion of a key role of lipid peroxidation mechanisms and/or JA signaling in plant responses to viruses.

The perception of specific pathogen-associated molecular patterns (PAMPs) by pattern recognition receptors (PRRs) activates initial defense responses and induces a downstream signaling cascade including phosphorylation events, a successive activation of cytoplasmic kinases (including the MAP kinases) and defense-related transcription factors and resistance genes. These mechanisms, well described for plant pathogenic bacteria, have been uncovered recently also in the case of plant responses to viruses [17, 42]. In our present study, we show that, in healthy BBP+ tomato plants, overexpression of systemin is able to induce the transcriptional activation of a set of RLKs that may act at the forefront of the pathogen perception mechanisms. *FLS2*, *HCR2-0A*, *SlRLK1* and *RLK-Solyc02g071810* were all transcriptionally activated in BBP+ plants. In tomato, systemin perception is dependent on a cell-surface receptor kinase named SR160 [4, 5], similar but not identical to BRI1, which on its turn can function as a systemin-binding protein [43]. In our study, we provide evidence that a wider set of receptor proteins respond as a direct or indirect result of systemin ectopic expression in tomato. Moreover, we show that some of these RLKs’ altered expression is positively or negatively correlated with the relieved symptoms induced by the two CMV/satRNA combinations in transgenic plants. In particular, higher expression levels of *LRR-RLK-Solyc04g076990*, *BRI1 and BAK1* and correlated with the suppression of symptoms observed in plants infected by FNNmut, whereas the lack of down-regulation of *FLS2* and *BRI1* or a reduced expression of *BAK1* and *SlRLK1* were peculiarly identified in more resistant (DN) vs. susceptible (RN) FN-infected plants.

An antiviral role of BAK1 has been shown in Arabidopsis plants, where it regulated PTI against three RNA viruses, namely *Tobacco mosaic virus* (TMV), *Oilseed rape mosaic virus* (ORMV) and *Tobacco crinkle virus* (TCV). In fact, *bak1* mutants showed an increased susceptibility to the three unrelated viruses during compatible interactions, and PTI markers were identified in crude plant extracts whose accumulation was BAK1-dependent [42]. The correlation that we found between higher *BAK1* expression and FNNmut-symptom suppression is consistent with these findings, although in our case a relevant up-regulation of *BAK1* also occurred in FN-infected plants showing RN versus those showing DN. However, none of the three viruses employed by Kørner and colleagues [42] induce necrosis. Observed differences may depend on cell death mechanisms that are expected to alter expression profiles of receptor proteins, including *BAK1*, differently than non-necrogenic damages [44, 45], and/or by additive effects of overexpressed systemin and virus-induced defense responses on RLKs’ expression levels.

In our experiments, *SlRLK1* showed an expression profile similar to that of *BAK1*, though more emphasized. We characterized here for the first time *SlRLK1* as the putative tomato orthologue of the pepper (*Capsicum annuum*) *CaRLK1*, which has been described as a negative regulator of plant cell death induced by *Xanthomonas campestris* pv. *vesicatoria* in both compatible and incompatible interactions, as well as by treatments with H_2_O_2_ or SA [46]. We observed an induction of *SlRLK1* upon CMV/satRNA infections significantly more pronounced in plants showing RN compared to those with DN (FN) or non-necrogenic symptoms (FNNmut). Overall, our data suggest that both *SlRLK1* and *BAK1* might function as basal regulators of host responses to FN-induced cell death in tomato.

The widely studied RLK FLS2 recognizes the flagellin of *Pseudomonas syringe* pv. *tomato* and other bacteria (e.g., *Xanthomonas* spp.), but not molecular patterns of fungal pathogens [47–50]. Although a role for FLS2 in antiviral responses was supposed to be unlikely in Arabidopsis [42], the same authors showed a decrease of *FLS2* expression levels and a concomitant overexpression of *BAK1* in leaf tissues at 21 dpi with ORMV. Therefore, their findings are not different from those presented in our work, and virus-induced suppression of RLKs such as *FLS2* might be part of a complex strategy adopted by viruses for repressing PTI-based plant immune responses. The first clear evidence of existence and efficacy of this viral strategy circumventing plant defenses has been published recently [51]. In this view, systemin-induced up-regulation of *FLS2*, *RLK-Solyc02g071810* and *LRR-RLK-Solyc04g076990*, all repressed by CMV/satRNA infections, may partially restore PTI-based responses and contribute to the reduced susceptibility observed in transgenic plants overexpressing *prosystemin*.

Finally, our results demonstrate that systemin plays a role in a compatible plant-virus interactions and has implications for several RLKs. Moreover, the ectopic overexpression of *prosystemin* in transgenic tomato is able to reinforce molecular defense barriers against CMV/satRNA infections by altering hormone-mediated and PTI-based basal responses. This increased readiness to respond to viral infections favored the non-necrogenic symptom suppression, whereas displayed a partial effectiveness against cell mechanisms leading to lethal necrosis.

## Supporting information

S1 Fig Symptoms induced by *Cucumber mosaic virus* (CMV) associated with its satellite RNA (satRNA) variants 21 days post-inoculation in tomato cv. Moneymaker plants. CMV-Fny, a severe strain belonging to the subgroup IA, causes reduced plant growth, mosaic and leaf malformations consisting in the so-called shoestring-leaf symptoms (leaf blade reduced to narrow strings corresponding to the midrib) or fern-leaf symptoms (the leaf blade is not completely suppressed as in shoestring, but is abnormally wrinkled and narrow). When CMV-Fny is associated with 77-satRNA, systemic necrosis develops rapidly, and when plantlets are inoculated at an early stage (i.e. at the four-leaf stage) the disease brings to death within three weeks ("lethal necrosis"). Three nucleotide substitutions in the necrogenic domain of 77-satRNA abolish its necrogenic nature and, hence, in this study were used to generate a non-necrogenic variant designated NNmut-satRNA. When CMV-Fny is co-inoculated with NNmut-satRNA symptoms of plant growth reduction (with shorter internodes), mosaic and leaf malformation (often milder that in the case of the helper virus alone), but not necrosis, occur in tomato.
(TIF)

S2 Fig Site-directed mutagenesis of 77-satRNA generates a non-necrogenic mutant satellite RNA. Alignment of 77-satRNA and NNmut-satRNA nucleotide sequences in a region including the necrogenic domain (in blue). Three nucleotide substitutions at positions 284, 289 and 291 (in red) within the necrogenic domain abolish the necrogenic nature of 77-satRNA (see S1 Fig for symptoms induced in tomato).
(TIF)

S1 Table List of RT-qPCR primers used in this study. (DOCX)
