# Supplementary figures and images for: Prosystemin overexpression induces transcriptional modifications of defense-related and receptor-like kinase genes and reduces the susceptibility to Cucumber mosaic virus and its satellite RNAs in transgenic tomato plants
Source: PLoS One. 2017 Feb 9;12(2):e0171902. doi: 10.1371/journal.pone.0171902 (PMC5300215; doi:10.1371/journal.pone.0171902)

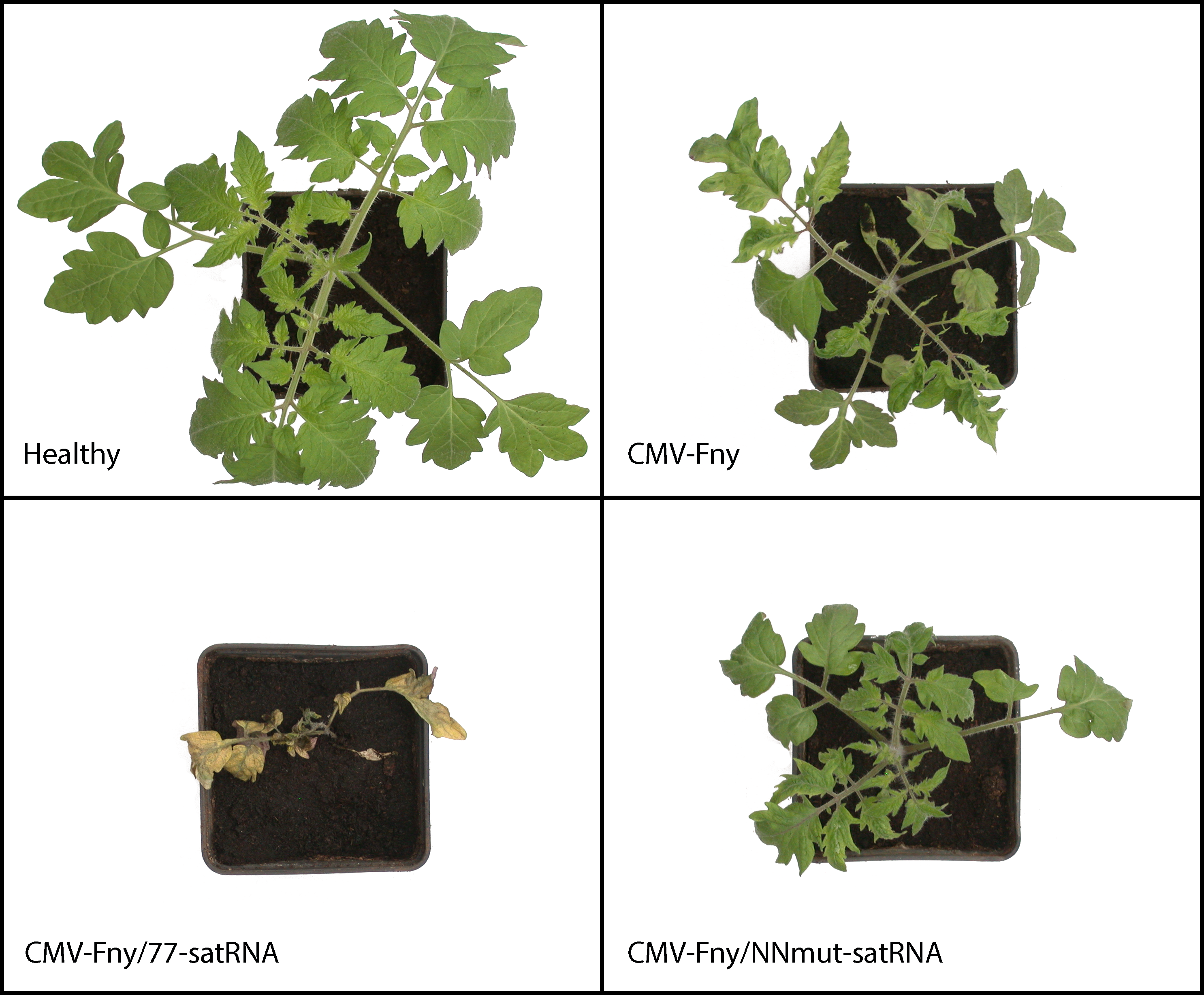

Supplement: S1 Fig — CMV-Fny, a severe strain belonging to the subgroup IA, causes reduced plant growth, mosaic and leaf malformations consisting in the so-called shoestring-leaf symptoms (leaf blade reduced to narrow strings corresponding to the midrib) or fern-leaf symptoms (the leaf blade is not completely suppressed as in shoestring, but is abnormally wrinkled and narrow). When CMV-Fny is associated with 77-satRNA, systemic necrosis develops rapidly, and when plantlets are inoculated at an early stage (i.e. at the four-leaf stage) the disease brings to death within three weeks ("lethal necrosis"). Three nucleotide substitutions in the necrogenic domain of 77-satRNA abolish its necrogenic nature and, hence, in this study were used to generate a non-necrogenic variant designated NNmut-satRNA. When CMV-Fny is co-inoculated with NNmut-satRNA symptoms of plant growth reduction (with shorter internodes), mosaic and leaf malformation (often milder that in the case of the helper virus alone), but not necrosis, occur in tomato. (TIF) [file pone.0171902.s001.tif]

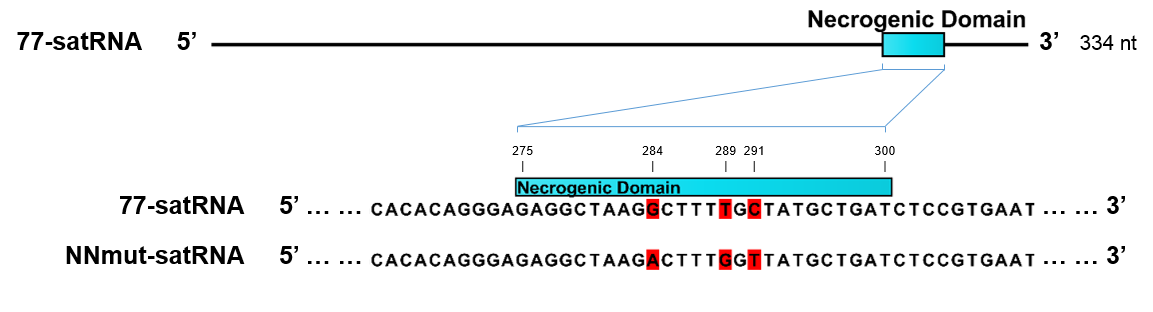

Supplement: S2 Fig — Alignment of 77-satRNA and NNmut-satRNA nucleotide sequences in a region including the necrogenic domain (in blue). Three nucleotide substitutions at positions 284, 289 and 291 (in red) within the necrogenic domain abolish the necrogenic nature of 77-satRNA (see S1 Fig for symptoms induced in tomato). (TIF) [file pone.0171902.s002.tif]
