# Supplementary material for: Prosystemin overexpression induces transcriptional modifications of defense-related and receptor-like kinase genes and reduces the susceptibility to Cucumber mosaic virus and its satellite RNAs in transgenic tomato plants
Source: PLoS One. 2017 Feb 9;12(2):e0171902. doi: 10.1371/journal.pone.0171902 (PMC5300215; doi:10.1371/journal.pone.0171902)
Supplement: S1 Table — (DOCX) [file pone.0171902.s003.docx]

S1 Table. List of RT-qPCR primers used in this study.

| **Name** | **GenBank accession, Sol Genomics Network locus or Unigene ID** | **Sol Genomics Network locus** | **Primer 5’->3’-sequences*^a, b^*** | **Amplicon length**  **(bp)** | **Reference** |
| --- | --- | --- | --- | --- | --- |
| ***Housekeeping gene*** | | | | | |
| UBI3 | X58253 Ubiquitin gene ubi3 |  | For: TCGTAAGGAGTGCCCTAATGCTGA  Rev: CAATCGCCTCCAGCCTTGTTGTAA | 119 | [1] |
| ***Cucumber mosaic virus*** | | | | | |
| CMV_RNA2 | NC_002035 *Cucumber mosaic virus*, RNA2, conserved region in *Fny* and *LS* strains |  | For: TGAGTTTGCTTGGTGTTATGACAC  Rev: CATCACCTTAGCTTCCATGTTGAA | 142 | [2] |
| ***Jasmonate pathway*** | | | | | |
| ProSys | M84801 Prosystemin |  | For: GGGAGGGTGCACTAGAAATA  Rev: TTGCATTTTGGGAGGATCAC | 110 | This study |
| loxD | U37840 Lipoxygenase-D |  | For: AAAGTTCATGGCCGTGGTTGACAC  Rev: ACAATCTCTGCATCTCCGGTCCAA | 105 | This study |
| AOS | AF230371 Allene oxide synthase |  | For: ACGGAAGAGCCAAACAGGACCTTA  Rev: CGTTGCAAATGGTTGGTACCCGAA | 98 | This study |
| Coi1 | AY423550 Coronatine-insensitive 1 |  | For: GAATTACTTGCGAATCGTCGTG  Rev: ACGAGAAATATGCAGAAGACCA | 96 | This study |
| PinI | K03290 Proteinase inhibitor I |  | For: TGAAACTCTCATGGCACGAAAAG  Rev: GGCCACATTTGTTTTCCTTCG | 102 | [3] |
| PinII | K03291 Proteinase inhibitor II |  | For: CCAAGAGGACCACTGAAAGA  Rev: ACACTCAGCCTCACGTAAA | 101 | [3] |
| ***SA pathway*** | | | | | |
| PR1b1 | NM_001247385 Pathogenesis-related protein 1b1 |  | For: CCAAGACTATCTTGCGGTTCA  Rev: CAGCTCTTGAGTTGGCATAGT | 113 | This study |
| PR-P2 | NM_001247154 Pathogenesis-related protein P2 precursor |  | For: AGAACTGCTAGCGTTTACTGCGCTAC  Rev: CCGCATGAAGCTTGGCCCGTAGGT | 119 | This study |
| ***Kinases*** | | | | | |
| FLS2 | SGN-U586739 Flagellin sensing 2 | Solyc02g070890.2.1 | For: TGGGAAAGCAAGAAGCTAAGGGCT  Rev: TGTTGGACAACGTCCAGTCATCCT | 106 | This study |
| BAK1 | NM_001247697 Somatic embryogenesis receptor kinase 3A (SERK3A) (or brassinosteroid insensitive 1-associated receptor kinase 1) | Solyc10g047140.1.1 | For: GAACGTCCAAAGATGTCAGAAG  Rev: GGTGGTGTACATGGTTGTAATC | 118 | [4] |
| BRI1 | SGN-U574182 Brassinosteriod-insensitive 1 (BRI1) | Solyc04g051510.1.1 | For: GGAAACGTCCCACAATGATAC  Rev: CCCTCCTTCAACTCCACTAA | 122 | [4] |
| HCR2-0A | SGN-U564475 Homologue of *Cladosporium* Resistance gene Cf-2 (Hcr2)-like | Solyc04g074030.2.1 | For: CGCGGTTACATTTGCTACCACTGA  Rev: TCCACTTAGCGTCCACGAAGCCAATA | 106 | This study |
| LRR-RLK | SGN-U586521 Leucine-rich repeat receptor-like kinase At1g09970-like | Solyc04g076990.2.1 | For: AGTGATGACTCGAGCTTGTTGGTG  Rev: ACCTCGTCTCCCAATCAAGTGACA | 103 | This study |
| RLK | Solyc02g071810 Receptor-like protein kinase | Solyc02g071810.2.1 | For: CCTCGCCCTTGCTTTATTT  Rev: GACGATTCTTCGTGTAGGAAAG | 117 | This study |
| SlRLK1 | Solyc05g009040 Receptor-like protein kinase | Solyc05g009040.2.1 | For: AAAGATCCGACAATGTTCGCCAA  Rev: GTGTTACAACCACGGTGCAAG | 100 | This study |

# References (for primer sequences listed above)

1. Mascia T, Santovito E, Gallitelli D, Cillo F. Evaluation of reference genes for quantitative reverse-transcription polymerase chain reaction normalization in infected tomato plants. Mol Plant Pathol. 2010;11(6):805-816. doi: 10.1111/j.1364-3703.2010.00646.x. PubMed PMID: 21029324.

2. Cillo F, Mascia T, Pasciuto MM, Gallitelli D. Differential effects of mild and severe *Cucumber* *mosaic* *virus* strains in the perturbation of microRNA-regulated gene expression in tomato map to the 3' sequence of RNA 2. MPMI 2009;22(10):1239-1249. doi: 10.1094/MPMI-22-10-1239. PubMed PMID: 19737097.

3. Tucci M, Ruocco M, De Masi L, De Palma M, Lorito M. The beneficial effect of *Trichoderma* spp. on tomato is modulated by the plant genotype. Mol Plant Pathol. 2011;12(4):341-354. doi: 10.1111/j.1364-3703.2010.00674.x. PubMed PMID: 21453429.

4. Zhang Z, Thomma BP. Structure-function aspects of extracellular leucine-rich repeat-containing cell surface receptors in plants. J Integr Plant Biol. 2013;55(12):1212-1223. doi: 10.1111/jipb.12080. PubMed PMID: 23718712.
